# The Effect of Dialectic Behavioral Counseling on Depression, Anxiety, and Postpartum Hematocrit Level

**DOI:** 10.1055/s-0041-1728780

**Published:** 2021-05-12

**Authors:** Nasrin Pour Amiri, Atefeh Ahmadi, Firoozeh Mirzaee, Moghadameh Mirzai, Nader Shahrokhi

**Affiliations:** 1 Kerman Nursing Research Center, Razi School of Nursing and Midwifery, Faculty of Kerman, University of Medical Sciences, Kerman, Iran

**Keywords:** postpartum depression, postpartum anxiety, hematocrit, dialectic behavioral therapy

## Abstract

**Objective** Childbirth is a biological, psychological, and sociological event that can be a positive or negative experience, and, without support, this period may be potentially damaging. Parturition may distort maternal emotions and lead to short- or long-term disorders such as postpartum depression and anxiety. The present research aims to study the effects of dialectic behavioral therapy-based counseling on depression, anxiety symptoms, and postpartum hematocrit level.

**Methods** The current research is a clinical trial study, and the sample was selected using parturients who were referred to the Health General Center with a diagnosis of postpartum depression and anxiety. The sample size consisted of 116 subjects who agreed to participate in the study. The patients in intervention group underwent group dialectic behavioral counseling (10 sessions/one session per week) and the control group did not receive any type of intervention. The patients were assessed in the first and last sessions as well as 2 months after the end of the sessions, using the Beck depression scale and Spielberg anxiety scale as well as the results of hematocrit tests. Data were analyzed using the IBM SPSS Statistics for Windows, Version 21.0 (IBM Corp., Armonk, NY, USA)

**Results** The results implied the effectiveness of dialectic behavioral therapy on reduction of the depression score, anxiety symptoms ( *p* -value ≤ 0.0001), and hematocrit level ( *p* -value = 0.04). The participants' depression, anxiety, and hematocrit levels decreased in the experiment group compared to the control group, and this decrease has remained until the 2-month follow-up.

**Conclusion** It seems that dialectic behavioral counseling reduces the levels of postpartum depression, anxiety, and hematocrits.

## Introduction

Pregnancy and postpartum bring significant psychological, physiological, and social changes which may sometimes come along with pathological changes, such as behavioral disorders. Women's mental health during pregnancy and in the postpartum period is important. Depression and anxiety are two of the psychological disorders that can happen during this period. They threaten the mother and child's health and are among the main health issues. The probability of occurrence of these disorders can be reduced when their effective factors are diagnosed and eliminated. 1

The prevalence of postpartum depression is 13 to 19% worldwide. Based on studies in other countries, the estimated postpartum depression rates reported in Hong Kong, China, South Africa, Turkey, Japan, and Brazil are 19.8%, 15.8%, 11.2%, 40.4%, 17% and 19.1%, respectively, and in Kerman and Iran, the rates are reported to be 20 to 30% and 31%, respectively. However, studies have concluded that during this period, the at-risk population is not diagnosed because many mothers do not want to be helped by others. 2 3 4

Postpartum blues is the most prevalent psychological issue. It can be described as a period of less than 2 weeks of mild depression symptoms that do not disturb the personal and social life of the mother. Postpartum depression, however, is the presentation of obvious sadness and/or symptoms of hopelessness, as intense as in a psychiatric disorder, for more than 2 weeks, according to American Psychiatry Association (APA). 5 6 The emergence of severe disorders (depression and anxiety) activates the immunity and inflammatory tracts, leading to a state of chronic inflammation and cellular activity in the immunity system. This process often causes secondary changes in the metabolism of iron and erythron, which reduces the hemoglobin, hematocrit, and iron levels in the blood. 7

If emerged, postpartum depression and anxiety cause issues for mother and infant as well as for other family members. Such states may impact the level of attachment of mother and as well as other familial relationships, and it may even negatively influence the child's development. Moreover, this causes behavioral, cognitive, social, and affective issues among infants which may continue up to ages 4 to 8. This may threaten the mother and child and other children's security and health. 6 7 8

Due to the possible complications of postpartum anxiety and depression and with the aim of obtaining a better prognosis, these disorders must be diagnosed early, and the treatment and therapeutic process must be initiated soon. Among the available therapeutic techniques used to treat these disorders, one can mention medication therapy; there is no absolute prohibition regarding the use of medications as the Food and Drug Administration (FDA) does not mention any specific medication that must be avoided during and after parturition. 9

The treatment for anxiety disorders and depression is mostly dependent on cognitive-behavioral therapy and psychotherapy, acceptance and commitment therapy, medication, and dialectic therapy or a combination of psychotherapy and medication therapy. Because of the possible side-effects of medications, they are used less than other techniques; therefore, these therapies are mostly effective, but not always. Consequently, for treating depression and anxiety disorders, a training program can help managing the disturbing ideas that cause dysfunction. 10

Dialectic behavioral counseling is a combination of supportive, cognitive, and behavioral therapies. This treatment is a novel therapeutic approach as one of third generation therapeutic theories which addresses skills in four elements of interventions in collective treatment, including mindfulness, interpersonal relationships efficiency, psychological skills, and distress tolerance skills. They are trained as suitable elements for different psychological diseases. 11 12 13 14 15 16

There have been numerous studies conducted in this regard. For example, Kleiber et al. (2017) 17 aimed to study the influence of dialectic behavioral counseling on depression and anxiety. This research did not measure the patients' hematocrit level, so the effect of this behavioral therapy on the hematocrit level was not analyzed. In Mashhad, Shafiee et al. (2017) 18 studied depression and anxiety symptoms by conducting a white and red blood cells count and a sexual-classified analysis in a cohort study. In this research, there was a decrease in the level of hematocrit in women with severe depression, but there was no postpartum study on women and no counseling used to reduce the symptoms. Martin et al. (2017) 19 studied mothers with persistent depression and severe emotional disorders treated with dialectic behavioral counseling. This research did not measure the participants' hematocrit level or the effects of counseling on hematocrit level. Khani et al. (2015) 20 studied the effect of dialectic behavioral therapy on depression score reduction. They didn't take anxiety into account because it is a phenomenon that generally accompanies depression. Generally, these studies did not study postpartum women. Based on our research, in Iran, there is no study that investigated the effect of dialectic behavioral counseling on postpartum depression, anxiety, and hematocrit level. Hence, the present work attempts to study these women who were referred to the Health General Center in Faryab city to receive postpartum healthcare.

## Methods

The present research is an experimental study with a pre and posttest and 2 months follow-up design with intervention and control groups. The population of parturients who were referred to the Health General Center for receiving postpartum healthcare was selected. The sample size selection was based on the available sampling procedure and inclusion criteria.

### Sample Size and Sampling Procedure

The statistical population consisted of parturients who had given birth 42 to 60 days prior to being referred to the Faryab City Health General Center for receiving postpartum healthcare. The sample size was calculated based on block randomization sampling and inclusion criteria.

### Inclusion Criteria

Having delivered a healthy child; having had a planned pregnancy; absence of immediate postpartum bleeding that required blood transfusion, and absence of chronic diseases in the mother, such as cardiovascular and endogenous glands diseases, before or after the pregnancy. 21 Minimum age of 18 years. 22 Having delivered a child between 42 and 60 days prior to inclusion in the study; 23 literacy; having signed the informed consent; being readily available for participation; presenting low-to-medium anxiety score based on the Spielberg anxiety scale (scores 20–60) as well as low depression score based on the Beck depression scale (scores 14–28). 24

### Exclusion Criteria

Significant stresses and unexpected events during the intervention. 25 26 Absence in more than 2 sessions and inability to follow relevant instructions and exercises as well as unwillingness to participate in the training and counseling sessions. 26 Mental retardation; menstruation bleeding; infections lasting longer than 7 days and that led to antibiotic taking. 21

### Data Collection

The Beck scale includes 21 multiple-choice (4 choices) items whose scores go from 0 to 3. Respondents can gain 63 scores, generally. In this scale, scores 0 to 13 indicate lack of depression, 14 to 28 indicate low depression, 29 to 44 indicate medium depression, and 45 to 62 indicate severe depression. 27 Respondents with low depression scores were included in the present study, and participants with medium and severe depression scores were referred to psychiatric care. The reliability and validity of this scale were measured in 1971, 1979, 1986, and 1986. This scale is of high validity. The internal consistency coefficient was measured to have an alpha coefficient of 0.81, and the overall validity coefficient was 0.91 for 21 items. The reliability of this questionnaire with the correlation coefficient with the retest method at a distance of one week is 0.94 and the correlation coefficient between the two halves is 0.89%.

### Spielberger State Inventory

The Spielberger state anxiety inventory is a widely used self-reported instrument. The Spielberger anxiety inventory asks respondents to report their anxiety levels during completion of the questionnaire. The items related to state anxiety in the Likert 4-choice spectrum are scored as never, very low, low, and relatively high. Each subject obtains 1 to 4 scores of each item. The scores domain is from 20 to 80 with 20 to 40 indicating subjects with low anxiety, 41 to 60 indicating subjects with medium anxiety, and 61 to 80 indicating subjects with severe anxiety. Subjects with low-to-medium anxiety were included in the study, and those with severe anxiety were excluded. 26 27 28 29 30

The reliability of this scale was reported by Spielberg et al. as being 0.83. This scale was normalized for the Iranian population with high validity and reliability. The reliability of the Spielberger state anxiety inventory was reported to be 0.04 using the retest technique at a 2-week interval. The alpha coefficient was 0.92, and the Pearson correlation coefficient was 0.75. 24 28

### Sample Size Calculation

Based on the following formula and using the recommendations of Roomruangwong et al. 7 and Parsa et al., 29 the sample size for this research was determined at 58 subjects for each of the groups (control and intervention).



Type 1 error: *α* = 0.05

Test power: 1- *β* = %80

Accuracy: *d* = 2



## Procedure

After receiving the permission from the ethics committee of the Kerman Medical Science University (IR.KMU.REC.1397.261) and taking the clinical tests, the authors referred to the Health General Centers selected subjects meeting the inclusion criteria and explained the procedure to them. After obtaining the signed informed consent, research scales (demographic information, Beck depression scale, and Spielberg anxiety scale) were completed by participants and a blood sample was taken to measure their hematocrit level. After analysis of the scales and calculation of scores, subjects with low depression and low-to-medium anxiety scores were included in the study. Individuals were assigned to groups by block randomization. Participants in the intervention group received dialectic behavioral therapy skills training with in-session exercises, homework, and debates each week in a 45-minute session for 10 weeks. The 10-session training included 3 sessions for personal emotion control, 3 sessions for distress tolerance, 3 sessions for interpersonal relationship development, and 1 session for mindfulness.

There were 5 to 10 participants in each session of the intervention group. After 10 sessions, the Beck and Spielberger scales were completed, and a blood sample was taken for hematocrit measurement in both the control and the experiment groups. Two months after the sessions ended, blood samples were collected to measure the hematocrit level, and the Beck and Spielberger scales were completed. Samples' collection took place from November 2017 to August 2018. Then, information of the Beck and Spielberger scales, demographic data, and hematocrit level were introduced into the IBM SPSS Statistics for Windows, Version 21.0 (IBM Corp., Armonk, NY, USA) and were analyzed.

## Results

The findings of the present research regarding demographic information indicated that the mean age of the participants, in both control and intervention groups, was 27 ± 6 years old, and there was no statistically significant difference between the groups. Both groups were homogenous in term of demographic variables, based on chi-squared and Fisher tests ( Table 1 ).

**Table 1:** Frequency distribution and percentages of demographic information in the intervention and control groups

Variable	Intervention group numbers (percentage)	Control group numbers (percentage)	*p* -value
Age			0.45
≥ 26	29 (29)	33 (56.9)	
˂ 26	29 (50)	25 (43.1)	
Education			0.39
High school	27 (46.6)	21 (36.2)	
Diploma	19 (32.8)	26 (44.8)	
Higher than diploma	1 (19)	12 (20.7)	
Number of pregnancies			0.6
1	20 (34.5)	17 (29.3)	
2	13 (22.4)	17 (29.3)	
3	12 (20.7)	15 (25.9)	
≥ 4	3 (22.4)	9 (15.5)	
Live children			0.88
1	29 (50)	25 (43.1)	
2	16 (27.6)	19 (32.8)	
3	8 (13.8)	19 (32.8)	
≥ 4	5 (8.6)	6 (10.3)	
Husband's job			0.23
Farmer	34 (58.6)	42 (72.4)	
Manual work	14 (24.2)	11 (19)	
Unemployed	10 (17.2)	5 (8.6)	
Income			0.88
˂ 1 million	24 (41.4)	24 (41.4)	
1–2 million	21 (36.2)	19 (32.8)	
2–3 million	13 (22.4)	15 (25.9)	
Child gender			0.22
Wanted	45 (77.6)	50 (86.2)	
Unwanted	13 (22.4)	8 (13.8)	
Abortion history			0.24
Yes	24 (41.4)	18 (31)	
No	34 (58.6)	40 (69)	
Delivery			0.15
NVD	37 (63.8)	44 (75.9)	
C/S	21 (63.2)	14 (24.1)	
Pregnancy			
Wanted	53 (91.4)	53 (91.4)	1
Unwanted	5 (8.6)	5 (8.6)	
Marriage			0.2
Wanted	57 (98.3)	53 (91.4)	
Unwanted	1 (1.7)	5 (8.6)	
Habitation			0.83
City	16 (27.6)	15 (25.9)	
Village	42 (72.4)	43 (74.1)	
Drug use			0.49
Yes	2 (3.4)	0	
No	56 (96.6)	58 (100)	
Drug abuse in husband			1
Yes	4 (6.9)	3 (5.2)	
No	54 (93.1)	55 (94.8)	
Problems in marital relations			0.28
Yes	6 (10.3)	10 (17.2)	
No	52 (89.7)	48 (82.8)	

Abbreviations: c/s, cesarean section; NVD, natural vaginal delivery.

Table 2 shows the mean comparison of the variables anxiety, depression, and hematocrit level before and after the intervention. According to the results of the intervention and control groups, the variables depression, anxiety and hematocrit level were homogeneous before the intervention.

**Table 2:** Comparison of the mean scores of anxiety, depression, and hematocrit levels in the intervention and control groups before intervention

Variables	Control groups	Intervention groups	*p* -value
Anxiety	38.37 ± 9.7	39.55 ± 8.7	0.49
Depression	15.94 ± 7.46	16.18 ± 7.43	0.86
Hematocrit	40.12 ± 3.42	40.37 ± 3.33	0.62

Based on Table 3 and the results of repeated measure test, the anxiety scores of the intervention group presented a reduction after the intervention and at the 2-month follow-up. In the control group. After this period, (10 weeks after the intervention and two months after the intervention in the intervention group), a small reduction was reported in the anxiety score. The control group did not receive any intervention. When comparing the depression scores, the intervention and control groups were significantly different ( *p* -value < 0.0001), that is, intervention reduced the depression scores in the intervention group.

**Table 3:** Comparison of mean scores of depression and anxiety and hematocrit level in the control and intervention groups after intervention and 2 months after it

		Group		*p* -value *
Variables		Control mean ± SD	Intervention mean ± SD	
**Anxiety**	After intervention	38.2 ± 9.59	29.32 ± 5.71	< 0.0001
	2 months follow-up	38.13 ± 9.76	25.67 ± 4.1	
**Depression**	After intervention	15.82 ± 7.49	7.18 ± 4.29	< 0.0001
	2 months follow-up	15.86 ± 7.32	4.89 ± 3.93	
**Hematocrit**	After intervention	40.09 ± 3.38	38.97 ± 2.94	0.04
	2 months follow-up	40.14 ± 3.44	38.23 ± 2.71	

Abbreviation: SD, standard deviation

* Adjusted for measurement before intervention

Based on Table 3 , there was a significant reduction in the depression scores after the intervention and at the 2-month follow-up in the intervention group, whereas in the control group, the reduction observed in the depression score was small. Comparing the intervention and the control groups, we concluded that interventions significantly reduced the depression scores in the intervention group compared to the control group ( *p* -value < 0.0001).

Based on Table 3 , the level of hematocrit in the intervention group before and after the intervention as well as in the 2-month follow-up decreased significantly. In the control group, when comparing the hematocrit level before and after the intervention, there was a reduction in the hematocrit level, followed by an increase at the 2-month follow-up. However, comparing both control and intervention groups in terms of hematocrit level, a statistically significant reduction was observed ( *p* -value = 0.04), that is, the intervention reduced the hematocrit level.

## Discussion

Depression and anxiety are problematic for the mother, the child, and other family members, as it may put the mother, the child, and other children's safety and health at risk, based on the increasing prevalence of depression and the necessity to study more effective approaches for treatment of this disorder. The present study aimed to determine the effects of dialectic behavioral therapy skills training on postpartum depression, anxiety symptoms, and hematocrit level.

In the present research, the anxiety scores were significantly reduced in the intervention group after the intervention, which is consistent with the results found by Akbarzadeh et al. (2012), 30 Rahimi et al. (2014), 31 and Bastani et al. (2005). 32

Moghadasi et al. (2016) 33 found that counseling reduces anxiety significantly. In a study by Muthusamy et al. (2012), 34 significant reduction of anxiety was observed in the intervention group compared with the control group.

In the study by Rahimi et al. (2014) 31 , anxiety remained unchanged in the control group after intervention, and, in a study by Moghadasi (2016), 33 anxiety reduction was reported in the control group after intervention, but all the results indicated that anxiety decreased significantly in the intervention group. 28 29 30 31 32 35 The decrease in the anxiety score reported in this research agree with the present results. Moreover, in the present study, partial reduction was reported in the control group, which was insignificant. The reason for this reduction may, based on what mothers reported in the control group, be easily used virtual networks from which they accessed information. Based on the findings of Ahmadi et al. (2014), 36 psychological services were influential on behavioral issues and postpartum anxiety.

The resent results are consistent with those of Khani et al. (2015). 20 They studied the effect of dialectic behavioral counseling on anxiety and found a decrease in the depression and anxiety scores. 20 In the present article, the results at the 2-month follow-up after the intervention were consistent with those of Khani et al. 20

In the study by Neacsiu et al. (2010) 13 , the effects of dialectic behavioral counseling on anxiety were studied, and they indicated significant reduction of depression and anxiety in the intervention group. Their results were consistent with ours, which can be due to similarities in how the counseling sessions were held, but in this study, there was no complete recovery among the participants due to lack of researchers' follow-up action for those with low depression and anxiety.

The study by Green et al. (2015) 37 investigated the cognitive-behavioral group therapy effects on prenatal period anxiety in an experimental design. This study was conducted on 10 parturients who received cognitive-behavioral therapy for 6 months until 12 months after parturition. The results indicated that participants reported increased acceptance and satisfaction with the therapeutic technique, and the findings implied that cognitive-behavioral therapy is a promising approach for reducing the prenatal period anxiety. Moreover, researchers found that this therapeutic technique is effective on depression symptoms. 37 The results of this study reported a significant reduction in the depression and anxiety scores after counseling, but the population of this research was small, and their counseling time was shorter than that of the present study. Moreover, the present study used a larger population with a 2-month follow-up action.

The present results were consistent with the results of Ahmadi et al. (2014), 36 who studied the effects of psychological services on postpartum depression.

Based on the results of Khani et al. (2015), 20 dialectic behavioral counseling was influential on the depression scores of the intervention group when compared to the control group. The present findings implied reduction of depression scores after dialectic behavioral counseling, consistent with Alizadeh et al. (2013). 38 The weaknesses of the study by Alizadeh et al. 38 are its small sample size and the lack of groups normalization as well as lack of follow-up for studying the persistence of therapy results.

In a research by Khadijeh et al. (2011), 39 dialectic behavioral skills training reduced depression. In the study by Meygoni and Ahadi (2012) 40 , patients with severe depression were studied, which implied significant difference in intervention group and behavioral therapy and medication therapy were compared. The results indicated that dialectic behavioral therapy significantly reduced the depression scores when compared to medication therapy. 40

This research was in agreement with the present work. This may be due to similarity of the therapeutic techniques and follow-up after the interventions. Maybe the weakness of this research was the pretest of depressed subjects in the first week after attempting suicide, which was highly effective on depression scores.

In a study by Sheydaei et al. (2017), 41 the partial effectiveness of mindfulness training on reducing postpartum depression symptoms was studied. The results indicated that mindfulness reduces the postpartum depression symptoms. 41 Mindfulness is important in dialectic behavioral therapy exercises, and it must be used in all sessions. Their results were consistent with ours, which may be due to shared mindfulness exercises used in both studies.

Lynch et al. (2015) 22 assessed and compared dialectic behavioral therapy, outpatient psychotherapy, and medication therapy and concluded that dialectic behavioral counseling is more effective that outpatient psychotherapy and medication therapy for persistent and chronic depression. This research was in full agreement with the present study in terms of counseling and follow-up after counseling.

In a study by Blackford and Love (2011), 42 the effects of dialectic behavioral therapy skills training were studied on mental health. They concluded that dialectic behavioral therapy skills training reduces the depression symptoms significantly and improves quality of life and social function among individuals. 42 The results of this research were consistent with ours in terms of counseling and effects on depression symptoms.

Kleiber et al. (2017) 17 studied the effects of behavioral therapy skills on mothers with prenatal depression (pregnancy and after parturition). They found that this skill reduces mothers' depression symptoms. 17 The results of this study were consistent with ours. In this research, the sessions and the homework were similar to ours, but there was no control group and none of the participants finished all sessions.

The results of the study by Martin et al. (2017) 19 on mothers with postpartum persistent depression and severe emotional disorders who received dialectic behavioral therapy skills training indicated that these skills reduced the mothers' depression symptoms and improve their parenting.

In a cohort study by Shafiee et al. (2017), 18 depression and anxiety symptoms were investigated by conducting a white and red blood cells count and a sexual-classified analysis. The results of this cohort study on 9,274 women and men indicated that Mean corpuscular volume (MCV) increases significantly among women with depression, but red blood cells and Hemoglobin (HB) decrease. Among women with severe anxiety, the Hematocrit (HCT), RBC, and Mean corpuscular hemoglobin concentration (MCHC) levels decreased, and among men with severe depression and anxiety symptoms, white blood cells and Red Cell Distribution Width (RDW) were higher than in women. 18 This research was inconsistent with the present study because they studied non-pregnant women with persistent or severe depression and anxiety, while the present study assessed parturients with low depression.

Roomruangwong et al. (2016) 7 studied the predictors of postpartum depression, anxiety symptoms, and hematocrit level. Their results were between predictors of postpartum depression and anxiety in spite of other studies. They found that factors such as depression before parturition and increase in the hematocrit level in the last 3 months of pregnancy are predictors of postpartum depression and anxiety symptoms. 7 These results were consistent with ours in terms of hematocrit increase. Based on the present results, it is implied that dialectic behavioral counseling reduced the anxiety and depression scores, and, as a result, the hematocrit level decreases among women with postpartum low-to-medium and low depression.

According to studied mentioned, there is no study rejecting the effects of dialectic behavioral counseling on mental disorders, and this may be due to familiarity with how to think in a healthy manner as well as with Linhan's maternal idea (the creator of this technique), which is mentioned throughout the therapeutic process.

Some participants were not willing to cooperate in this study due to mental issues resulting from depression and anxiety or due to lack of their sexual partner's agreement. To overcome these issues, the wife and husband were interviewed in private. Lack of patience for participating in the sessions was another issue, which was eliminated after the goals of study were revealed and the therapeutic technique was explained and mindfulness was mostly focused. The participants' depression and anxiety scores were assessed based on scales. We recommend that in future studies, a clinical interview by is conducted by a psychiatrist based on the fourth edition of the Diagnostic and Statistical Manual of Mental Disorders (DSM-IV)

## Conclusion

Based on the results obtained, early intervention with dialectical behavior therapy skills training for women with pregnancy and postpartum-related depression and anxiety can reduce depression, anxiety, and hematocrit level. Since the mental health of mothers in the postpartum period is very important, health care systems should consider related psychological interventions.
